# Pace-Induced Saccades in Essential Tremor Differ from Those in Parkinson’s Disease and Degenerative Ataxias

**DOI:** 10.3390/jcm15114054

**Published:** 2026-05-24

**Authors:** Magdalena Wójcik-Pędziwiatr, Monika Rudzińska-Bar

**Affiliations:** Department of Neurology, Faculty of Medicine, Collegium Medicum, Andrzej Frycz Modrzewski Krakow University, 30-705 Kraków, Poland

**Keywords:** essential tremor, Parkinson’s disease, degenerative ataxias, eye movements, saccades, oculomotor abnormalities, differential diagnosis, pace-induced saccades, ET-plus

## Abstract

**Background:** Essential tremor (ET) is increasingly recognized as a heterogeneous disorder that may present with additional Parkinsonian or cerebellar features, complicating its differential diagnosis from Parkinson’s disease (PD) and degenerative ataxias (DA). Oculomotor assessment has been proposed as a potential tool for identifying disease-specific patterns. **Methods:** We evaluated eye movement parameters in 50 patients with ET (including subgroups with Parkinsonian and/or cerebellar signs), 50 patients with PD, 42 patients with DA, and 42 healthy controls. Reflexive, pace-induced, and cued saccades were recorded using saccadometry, while smooth pursuit and fixation were assessed with electrooculography. Group comparisons focused on saccadic metrics and the frequency of abnormalities. **Results:** Hypometria of pace-induced saccades was observed in patients with PD (32.0%) and DA (57.1%) but was not detected in any ET subgroup, including those with Parkinsonian or cerebellar features. Reflexive saccade hypometria was less frequent in ET patients with Parkinsonian features compared to PD (50.0% vs. 66.0%, *p* = 0.016). Other oculomotor parameters showed substantial overlap between groups. **Conclusions:** ET patients, including those with additional Parkinsonian or cerebellar signs, showed partially distinct oculomotor features compared with PD and DA, particularly in pace-induced saccades. These findings require confirmation in larger, well-characterized cohorts.

## 1. Introduction

Essential tremor (ET) has traditionally been considered a monosymptomatic movement disorder characterized primarily by action tremor. However, accumulating clinical and neurophysiological evidence suggests that ET represents a heterogeneous condition that may include a broad spectrum of additional motor and non-motor features. These may encompass Parkinsonian signs, cerebellar dysfunction, cognitive impairment, and neuropsychiatric symptoms, challenging the classical view of ET as a benign and isolated entity [1,2]. In particular, the presence of Parkinsonian or cerebellar signs in patients with ET may complicate clinical diagnosis and lead to potential misclassification as Parkinson’s disease (PD) or degenerative ataxias [3].

The differential diagnosis between ET, PD, and cerebellar ataxias remains a significant clinical challenge, especially in patients presenting with overlapping or atypical features. Epidemiological studies suggest that a substantial proportion of patients initially diagnosed with ET are later reclassified as having alternative movement disorders [4]. Moreover, the relationship between ET and PD remains incompletely understood, with some evidence indicating a possible pathophysiological overlap or increased risk of PD in subsets of ET patients [5,6]. Similarly, cerebellar involvement in ET has been increasingly recognized, supported by clinical findings, neuroimaging studies, and postmortem data [7].

Oculomotor assessment has emerged as a valuable tool in the investigation of neurological disorders, providing objective and quantifiable measures of central nervous system function. Eye movement abnormalities, including deficits in saccades, smooth pursuit, and fixation, have been described in a range of neurodegenerative conditions [8,9]. In PD, characteristic findings include hypometria, increased latency, and reduced saccade velocity, particularly in voluntary paradigms [10,11]. In contrast, degenerative ataxias are typically associated with a broader spectrum of oculomotor disturbances, reflecting dysfunction of cerebellar and brainstem circuits [12]. Previous studies have suggested that oculomotor testing may aid in the differential diagnosis of these conditions and may serve as a potential biomarker of disease-specific neural dysfunction [13].

The role of oculomotor abnormalities in ET remains less well defined. While earlier studies suggested relative preservation of eye movements in ET, more recent investigations have demonstrated subtle impairments, particularly in patients with prominent cerebellar features [14,15,16]. Abnormalities, such as saccadic dysmetria, prolonged latency, and impaired smooth pursuit, have been reported, supporting the involvement of cerebellar and cortico-subcortical networks in the pathophysiology of ET [16]. However, data regarding ET patients presenting with additional Parkinsonian signs are scarce. This subgroup may represent a diagnostically challenging phenotype, potentially overlapping with the concept of “ET-plus” as proposed in recent tremor classifications [17].

Recent studies have highlighted the growing role of eye-tracking as a non-invasive biomarker in neurodegenerative diseases; however, its diagnostic specificity remains limited [18,19,20].

Despite growing interest in oculomotor abnormalities and eye-tracking biomarkers in movement disorders, studies directly comparing patients with ET presenting with Parkinsonian or cerebellar features with well-defined cohorts of PD and degenerative ataxias using standardized eye movement paradigms remain limited. Moreover, the clinical interpretation of oculomotor abnormalities in ET-spectrum disorders remains uncertain, particularly in patients with overlapping Parkinsonian or cerebellar features. This diagnostic uncertainty reflects the evolving understanding of ET as a heterogeneous spectrum disorder and highlights the need for clinically interpretable oculomotor paradigms. In particular, the diagnostic value of voluntary saccade paradigms, such as pace-induced and cued saccades, in differentiating these conditions has not been fully elucidated.

The present study aimed to compare oculomotor parameters among patients with ET, including subgroups with Parkinsonian and/or cerebellar signs, patients with PD, and patients with degenerative ataxias. We hypothesized that patients with ET exhibiting additional neurological signs would demonstrate partially overlapping but distinguishable oculomotor profiles compared to those with PD and degenerative ataxias, particularly in voluntary saccade paradigms. By identifying specific patterns of eye movement abnormalities, this study seeks to contribute to the understanding of the pathophysiology of ET and evaluate the potential clinical utility of oculomotor testing in the differential diagnosis of movement disorders.

## 2. Materials and Methods

### 2.1. Study Design and Participants

This cross-sectional observational study included patients diagnosed with essential tremor (ET), Parkinson’s disease (PD), and degenerative ataxias (DA), as well as healthy control subjects. Participants were recruited from the Movement Disorders Clinic, Department of Neurology, University Hospital in Krakow, Poland, between 2010 and 2014.

A total of 50 patients with ET, 50 patients with PD, 42 patients with degenerative ataxias, and 42 healthy controls were enrolled. The control group was matched to the patient groups by age (±3 years) and sex. All participants provided written informed consent before inclusion in the study.

### 2.2. Diagnostic Criteria and Clinical Assessment

The diagnosis of ET was established according to the National Institutes of Health Collaborative Genetic Criteria (1996), while PD was diagnosed based on the UK Parkinson’s Disease Brain Bank Criteria. The diagnosis of degenerative ataxias was based on clinical presentation supported by brain magnetic resonance imaging and, where available, genetic testing.

All participants underwent a structured clinical assessment including medical history, neurological examination, and evaluation of disease characteristics such as age at onset, disease duration, and symptom profile. Cognitive status was assessed using the Mini-Mental State Examination (MMSE), and depressive symptoms were evaluated with the Beck Depression Inventory (BDI).

Disease severity was assessed using validated clinical scales: the Clinical Rating Scale for Tremor (CRST) for ET patients, the Unified Parkinson’s Disease Rating Scale (UPDRS) for PD patients, and the International Cooperative Ataxia Rating Scale (ICARS) for patients with degenerative ataxias.

### 2.3. Subgroup Classification

Patients with ET were further categorized into subgroups based on the presence of additional neurological signs identified during clinical examination:

- ET-T, isolated tremor;
- ET-P, tremor with Parkinsonian signs;
- ET-C, tremor with cerebellar signs;
- ET-M, tremor with both Parkinsonian and cerebellar signs.

Parkinsonian signs included resting tremor, bradykinesia, and rigidity, while cerebellar signs included intention tremor, dysdiadochokinesia, and gait disturbances.

Exclusion criteria for all participants included:

- restriction of eye movements or significant ophthalmological disorders (e.g., scotoma, severe refractive errors, color blindness),
- neurological or neuromuscular disorders affecting oculomotor function other than the studied conditions,
- use of medications known to significantly affect eye movements (except levodopa, propranolol, and primidone),
- history of substance abuse or toxic exposure,
- severe systemic illness (cardiac, renal, hepatic, or pulmonary),
- psychiatric disorders such as schizophrenia,
- endocrine disorders affecting neurological function (e.g., hypo- or hyperthyroidism),
- contraindications to MRI.

Eligibility was verified through clinical evaluation, laboratory testing (including TSH and ceruloplasmin), ophthalmological examination, and neuroimaging where appropriate.

### 2.4. Oculomotor Assessment

Eye movements were evaluated using a combination of saccadometry and electrooculography (EOG). All recordings were conducted in the morning, prior to the administration of the participants’ regular medications. Participants were instructed to avoid alcohol and caffeine for at least 24 h before testing. Patients with PD were examined in the practically defined OFF state.

Examinations were performed in a quiet, dimly lit room. Participants were seated in a comfortable chair with head stabilization and positioned at a fixed distance of 1 m from a projection screen.

#### 2.4.1. Saccadic Eye Movements Assessment

Saccadic eye movements were recorded using the Saccadometer Advanced system (Ober Consulting, Poznań, Poland). The following paradigms were assessed:

##### Reflexive Saccades Assessment

Reflexive saccades were used to evaluate latency, amplitude, and velocity. Participants were instructed to shift their gaze from a central fixation point to a peripheral target as soon as it appeared.

Saccade amplitude was considered normal if it reached 85–100% of the target distance (17–20° for 20° saccades). Dysmetria was defined as a frequency of hypo- or hypermetric saccades exceeding 23% [14].

##### Pace-Induced Saccades Assessment

In the pace-induced paradigm, two peripheral targets were continuously displayed for 30 s, during which participants were instructed to alternately shift their gaze between them as quickly and accurately as possible. The following parameters were analyzed: number of saccades, latency, amplitude, velocity, and frequency of dysmetria.

##### Cued Saccades Assessment

Cued saccades were evaluated using a color-based paradigm in which participants were instructed to direct their gaze toward a peripheral target based on the color of a central cue (green or red). Outcome measures included the error rate (incorrect direction) and latency of correct responses.

Each paradigm included calibration trials followed by 100 experimental trials. Data were analyzed using LatencyMeter software (version 4.11, Ober Consulting, Poland).

#### 2.4.2. Smooth Pursuit and Fixation Assessment

Smooth pursuit and fixation were assessed using conventional electrooculography (EOG). EOG activity was recorded using surface electrodes placed at the lateral canthi of both eyes to measure horizontal eye movements. A reference (ground) electrode was positioned on the forehead. Smooth pursuit gain was calculated as the ratio of eye velocity to target velocity. Eye movements were recorded using electrooculography during sinusoidal target motion.

The target position followed a sinusoidal trajectory, as follows: x(t) = Asin(2πft).

In the present study, the stimulus frequency was 0.2 Hz, and the amplitude was ±15°. The target velocity was analytically derived from the stimulus parameters, with the peak velocity defined as 2πfA.

Eye position signals were digitally filtered and differentiated to obtain the eye velocity. Saccades were identified using a velocity threshold criterion and excluded from further analysis. Only the smooth-pursuit component was retained.

Gain was calculated as: gain = V_eye_/V_target_.

For each trial, gain was computed as the ratio of peak eye velocity to peak target velocity (velocity gain). Additionally, instantaneous gain was calculated sample by sample and averaged over the stimulus cycle after removal of saccadic segments.

Gain values were analyzed separately for leftward and rightward pursuit and averaged across cycles. For each participant, the final gain value was calculated as the mean of four consecutive measurements.

Visual fixation was assessed using electrooculography (EOG) with the same electrode configuration as for smooth pursuit recordings. Subjects were instructed to maintain a steady gaze on a stationary visual target positioned at eye level. Recordings were performed under constant illumination, and fixation stability was evaluated based on the presence of ocular drifts, square-wave jerks, and other intrusive saccadic movements.

### 2.5. Statistical Analysis

Statistical analyses were performed using STATISTICA software (version 9.0, StatSoft Inc., Kraków, Poland). Continuous variables are presented as mean ± standard deviation (SD). Normality of distribution was assessed using the Kolmogorov–Smirnov test.

Comparisons between two groups were performed using the Mann–Whitney U test for continuous variables and the χ^2^ test (with Yates correction when appropriate) or Fisher’s exact test for categorical variables. For comparisons involving more than two groups, the Kruskal–Wallis test was used, followed by Dunn’s post hoc test. Given the exploratory nature of the study and the relatively small subgroup sizes, formal correction for multiple comparisons was not applied, and the results should therefore be interpreted cautiously and considered hypothesis-generating.

A *p*-value < 0.05 was considered statistically significant.

## 3. Results

### 3.1. Study Population

A total of 184 participants were included in the study: 50 patients with essential tremor (ET), 50 patients with Parkinson’s disease (PD), 42 patients with degenerative ataxias (DA), and 42 healthy controls. The control group consisted of healthy volunteers without neurological or psychiatric disorders, matched for age and sex to the patient groups. None of the controls were taking medications affecting the central nervous system.

The general clinical characteristics of ET, PD, and DA groups are presented in Table 1, while detailed characteristics of ET subgroups are summarized in Table 2.

Within the ET cohort, patients were classified into four subgroups: ET-T (isolated tremor), ET-P (Parkinsonian signs), ET-C (cerebellar signs), and ET-M (mixed phenotype).

#### Clinical Characteristics of ET Subgroups

ET subgroups differed in clinical profile, reflecting the heterogeneity of the disorder. Parkinsonian features were present in the ET-P and ET-M groups, whereas cerebellar signs predominated in ET-C and ET-M. All patients in the ET-P and ET-M groups exhibited at least one Parkinsonian sign, while all patients in the ET-C and ET-M groups presented cerebellar features (Table 2).

There were no significant differences in age or age at disease onset between ET subgroups. In contrast, disease duration and tremor severity differed significantly across groups. Patients with isolated tremor (ET-T) had shorter disease duration and lower CRST scores compared with other ET subgroups.

Values are presented as mean ± SD or number (percentage). Statistical comparisons were performed using the Kruskal–Wallis test for continuous variables. No statistical comparisons were performed for categorical clinical features; these data are presented descriptively.

### 3.2. Oculomotor Findings

A comprehensive summary of oculomotor parameters across all study groups is presented in Table 3.

#### 3.2.1. Reflexive Saccades Performance

No significant differences in latency, amplitude, or velocity of reflexive saccades were observed between ET patients and controls.

**Figure 1:**
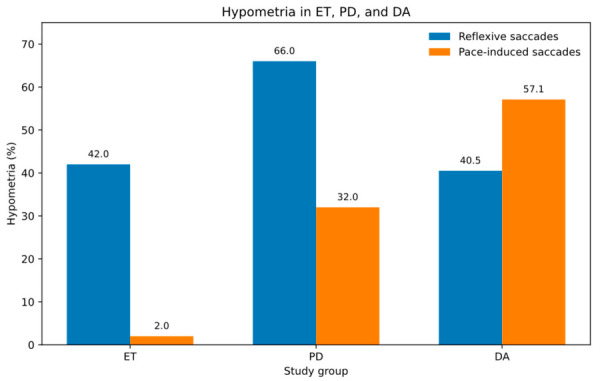
Hypometria of reflexive and pace-induced saccades across study groups. The frequency of hypometria in reflexive and pace-induced saccades is shown for patients with essential tremor (ET), Parkinson’s disease (PD), and degenerative ataxias (DA). ET values represent pooled data from all ET subgroups. Hypometria of pace-induced saccades was observed in PD and DA but was virtually absent in ET, whereas reflexive saccade hypometria showed substantial overlap between groups. The absence of pace-induced saccadic hypometria in ET subgroups represented the most consistent differentiating feature.

Hypometria of reflexive saccades was more frequent in PD patients compared to ET patients with Parkinsonian features (66.0% vs. 50.0%, *p* = 0.016), while values in other ET subgroups remained comparable to controls. This difference is illustrated in Figure 1. No significant differences were observed between ET patients with cerebellar signs and patients with degenerative ataxias.

#### 3.2.2. Pace-Induced Saccades Performance

The most prominent differences between groups were observed in the pace-induced saccade paradigm (Table 3).

Hypometria of pace-induced saccades was present in patients with PD (32.0%) and degenerative ataxias (57.1%) but was not detected in any ET subgroup, including those with Parkinsonian or cerebellar features.

Additionally, saccade amplitude was reduced in patients with degenerative ataxias compared with ET-C patients (*p* = 0.025). Latency and velocity parameters did not differ significantly between groups.

#### 3.2.3. Cued Saccades Performance

In the cued saccade paradigm, no consistent differences were observed between ET subgroups and other disease groups.

Although PD patients showed a tendency toward higher error rates, these differences did not reach statistical significance.

#### 3.2.4. Smooth Pursuit and Fixation Performance

Smooth pursuit gain was lowest in PD patients and reduced in degenerative ataxias, whereas ET subgroups showed intermediate values without significant differences.

Fixation stability was largely preserved in ET patients, whereas fixation instability and saccadic intrusions were more frequently observed in PD and degenerative ataxias.

#### 3.2.5. Summary of Oculomotor Findings

Overall, oculomotor findings showed substantial overlap across groups, with the most notable differences observed in the pace-induced saccade paradigm.

## 4. Discussion

This study investigated oculomotor function in patients with essential tremor (ET), including subgroups with Parkinsonian and cerebellar signs, in comparison with those with Parkinson’s disease (PD) and degenerative ataxias (DA). The main finding was the observation of hypometria of pace-induced saccades in patients with PD and DA but not in those with ET, even in those presenting with additional neurological signs. In contrast, other oculomotor parameters showed substantial overlap between the groups, suggesting a limited overall diagnostic utility of oculomotor testing in this clinical context.

### 4.1. Oculomotor Profiles in ET, PD, and Degenerative Ataxias

Eye movement abnormalities have been extensively described in patients with PD and cerebellar ataxias, reflecting dysfunction within distinct neural circuits. In PD, deficits in voluntary saccades, including hypometria and increased latency, are commonly attributed to dysfunction of the frontobasal ganglia–superior colliculus pathways [8,10,11]. In contrast, degenerative ataxias are typically associated with impaired accuracy and coordination of eye movements due to cerebellar involvement, often resulting in dysmetria, impaired smooth pursuit, and fixation instability [12,13]. Recent studies have further emphasized the growing interest in oculomotor markers as potential biomarkers of neurodegenerative processes [18,19].

In line with previous studies, our findings confirm the presence of saccadic hypometria in PD and degenerative ataxias, particularly in voluntary paradigms. The observation that hypometria of pace-induced saccades was absent in patients with ET, including those with Parkinsonian or cerebellar signs, is noteworthy. This may suggest that the neural mechanisms underlying voluntary saccade control are relatively preserved in ET compared to PD and DA. However, given the complexity of oculomotor control, this interpretation should be considered with caution.

### 4.2. Essential Tremor as a Heterogeneous Disorder

The results of this study support the growing body of evidence that ET is a heterogeneous condition with variable clinical expression. The presence of additional neurological signs has been incorporated into recent tremor classifications under the concept of “ET-plus” [17]. These phenotypes pose a diagnostic challenge, as they may clinically overlap with PD or cerebellar syndromes. Interestingly, despite the presence of such features, the ET subgroups in our study did not demonstrate oculomotor patterns characteristic of PD or degenerative ataxias. This finding may indicate that these additional features do not necessarily reflect the same underlying pathophysiological mechanisms as in primary PD or cerebellar degeneration. The clinical interpretation of ET subgroups presenting with additional Parkinsonian or cerebellar features remains challenging, particularly in light of the evolving concept of ET-plus and the recognized overlap between tremor syndromes and early neurodegenerative disorders. Some patients classified as ET-P or ET-C may represent phenotypic overlap cases or early stages of Parkinson’s disease or cerebellar degeneration. However, subgroup classification in the present study was based on detailed neurological examination and reflected the exploratory and clinically oriented design of the study rather than biomarker-based diagnostic categorization. These findings further support the concept of ET as a heterogeneous spectrum disorder with variable clinical expression. Alternatively, it is possible that oculomotor testing, as applied in this study, lacks sufficient sensitivity to detect subtle differences between these conditions.

### 4.3. Diagnostic Utility of Oculomotor Testing

A key clinical question addressed in this study was whether oculomotor assessment could aid in the differential diagnosis of ET, PD, and degenerative ataxias. Although certain differences were identified, notably in pace-induced saccades, the overall overlap between groups was substantial. These findings may reflect differences in underlying network dysfunction. However, the substantial overlap between groups suggests that oculomotor assessment has limited utility as a standalone diagnostic tool, consistent with recent literature highlighting both the potential and limitations of eye movement biomarkers in clinical practice [18,19,20].

Notably, the absence of hypometria in pace-induced saccades across all ET subgroups represents the most consistent differentiating feature identified in this study. Although the data were collected between 2010 and 2014, the oculomotor paradigms used in the present study remain based on established neurophysiological principles that continue to be applied in contemporary eye movement research. The primary aim of this study was to compare characteristic patterns of oculomotor abnormalities across clinically defined movement disorder groups rather than to evaluate technology-dependent biomarkers.

### 4.4. Pathophysiological Considerations

From a pathophysiological perspective, voluntary saccades are mediated by a distributed network involving the frontal cortical areas, basal ganglia, superior colliculus, and cerebellum. In PD, dysfunction within basal ganglia circuits is thought to impair the initiation and scaling of voluntary saccades, leading to hypometria. In contrast, cerebellar ataxias primarily affect movement accuracy and coordination. The relative preservation of pace-induced saccade accuracy in ET may indicate a different pattern of network involvement compared to PD and degenerative ataxias. Therefore, it is possible that the abnormalities present in ET are more subtle, variable, or task-dependent than those captured by the paradigms used in this study.

### 4.5. Comparison with Previous Studies

Our findings are consistent with earlier reports demonstrating mild or inconsistent oculomotor abnormalities in ET compared to more pronounced deficits in PD and cerebellar ataxias. Helmchen et al. reported cerebellar-type oculomotor abnormalities in ET, particularly in patients with more severe disease [14]. Similarly, Visser et al. identified differences in saccadic parameters between ET and tremor-dominant PD, although with considerable overlap [16].

Few studies have specifically addressed patients with ET who have additional Parkinsonian or cerebellar features. The present study extends previous work by focusing on these clinically relevant subgroups, highlighting the complexity of ET phenotypes and the challenges in differentiating them from other movement disorders.

### 4.6. Limitations

Several limitations of this study should be acknowledged. First, the number of patients in certain ET subgroups, particularly those with Parkinsonian signs (ET-P and ET-M), was relatively small, which may have limited the statistical power and generalizability of subgroup analyses. Therefore, these findings should be interpreted cautiously and require confirmation in larger cohorts. Second, the saccadometer system records only the first saccade and may not fully capture more complex saccadic behavior, including corrective movements. Third, the DA cohort was clinically heterogeneous and included different cerebellar syndromes that may affect oculomotor control through partially distinct mechanisms. Some cerebellar ataxias may be associated predominantly with hypometric saccades, whereas others may produce hypermetric patterns, potentially increasing variability in the observed oculomotor parameters. However, the DA cohort was not further subdivided into specific diagnostic categories because no clear differentiating clinical or oculomotor features were identified during the performed evaluations. All patients presented with cerebellar syndrome on neurological examination and demonstrated radiological features of cerebellar atrophy on neuroimaging studies, while genetic testing for the most common spinocerebellar ataxias (SCA1, SCA2, and SCA3) was negative in all tested patients.

Additionally, the data were collected between 2010 and 2014, and both diagnostic concepts and eye-tracking methodologies have evolved since that time. In particular, contemporary classifications such as the concept of ET-plus were introduced after the original data collection period. Furthermore, the classification of ET subgroups was based primarily on clinical examination and was not supported by biomarker or longitudinal validation, which may carry a risk of phenotypic overlap or misclassification bias, particularly with prodromal Parkinson’s disease or early cerebellar syndromes. Therefore, retrospective interpretation of some clinical phenotypes should be approached with caution, although the analyzed oculomotor paradigms remain clinically and physiologically relevant.

In addition, the analyses were limited to univariate comparisons and did not adjust for potential confounding factors such as age, disease duration, or disease severity. Given the relatively small subgroup sizes, multivariate modeling was considered potentially unstable and at risk of overfitting. Moreover, multiple comparisons were performed without formal correction; therefore, the findings should be considered exploratory and hypothesis-generating. Finally, although efforts were made to standardize the testing conditions, assessment of certain parameters, such as fixation stability, included a qualitative component that may have introduced observer-related variability.

### 4.7. Clinical Implications and Future Directions

Despite these limitations, the present study provides clinically relevant insights into oculomotor function in ET and related disorders. Future studies should include larger cohorts and incorporate advanced eye-tracking techniques, including machine learning-based approaches, to further improve diagnostic accuracy [20,21,22,23]. The integration of oculomotor data with neuroimaging, neurophysiological, and computational approaches may further enhance our understanding of disease-specific mechanisms and improve diagnostic accuracy.

## 5. Conclusions

In summary, patients with ET, including those presenting with additional Parkinsonian or cerebellar signs, showed partially distinct oculomotor features compared with PD and degenerative ataxias, particularly in pace-induced saccade paradigms. However, the substantial overlap between groups limits the clinical utility of oculomotor assessment as a standalone diagnostic tool. Therefore, these findings should be interpreted cautiously and considered exploratory. Further studies in larger, well-characterized cohorts are needed to better define the role of eye movement analysis in movement disorders and to further characterize the heterogeneity of ET.

## Figures and Tables

**Table 1:** Demographic and clinical characteristics of study groups.

Parameter	ET (*n* = 50)	PD (*n* = 50)	DA (*n* = 42)	Controls (*n* = 42)
Age (years)	59.2 ± 21.7	64.9 ± 11.3	55.2 ± 10.5	60.6 ± 19.0
Male, n (%)	26 (52%)	27 (54%)	24 (57%)	25 (50%)
Age at onset (years)	40.1 ± 21.3	55.0 ± 12.0	38.1 ± 15.5	–
Disease duration (years)	19.4 ± 12.4	11.4 ± 4.1	12.1 ± 6.3	–
CRST score	30.9 ± 15.0	–	–	–
UPDRS score	–	30.3 ± 13.9	–	–
ICARS score	–	–	23.5 ± 6.3	–
MMSE score	28.1 ± 1.9	27.9 ± 2.7	28.3 ± 2.5	–
BDI score	11.7 ± 9.8	13.1 ± 12.4	8.2 ± 10.2	–

Abbreviations: ET, essential tremor; PD, Parkinson’s disease; DA, degenerative ataxias; CRST, Clinical Rating Scale for Tremor; UPDRS, Unified Parkinson’s Disease Rating Scale; ICARS, International Cooperative Ataxia Rating Scale; MMSE, Mini-Mental State Examination; BDI, Beck Depression Inventory. Values are presented as mean ± SD or number (percentage).

**Table 2:** Clinical characteristics of essential tremor (ET) subgroups.

Parameter	ET-T (*n* = 17)	ET-P (*n* = 6)	ET-C (*n* = 20)	ET-M (*n* = 7)	*p*-Value
**Age (years)**	48.1 ± 23.0	64.8 ± 11.0	64.5 ± 21.1	65.0 ± 21.1	ns
**Age at onset (years)**	36.9 ± 21.5	39.8 ± 24.7	42.7 ± 21.6	40.8 ± 20.4	ns
**Disease duration (years)**	16.1 ± 31.0	29.4 ± 16.7	21.1 ± 13.0	25.5 ± 9.7	*p* = 0.002
**CRST score**	20.7 ± 11.9	32.7 ± 8.5	33.3 ± 12.5	43.1 ± 18.0	*p* = 0.002
**Parkinsonian signs**					
Rest tremor	0 (0%)	5 (83.3%)	0 (0%)	4 (57.1%)	
Bradykinesia	0 (0%)	4 (66.7%)	0 (0%)	4 (57.1%)	
Rigidity	0 (0%)	4 (66.7%)	0 (0%)	2 (28.6%)	
**Any Parkinsonian sign**	0 (0%)	6 (100%)	0 (0%)	7 (100%)	
**Cerebellar signs**					
Intention tremor	0 (0%)	0 (0%)	14 (70.0%)	6 (85.7%)	
Dysdiadochokinesia	0 (0%)	0 (0%)	6 (30.0%)	3 (42.9%)	
Gait disturbance	0 (0%)	0 (0%)	4 (20.0%)	1 (14.3%)	
**Any cerebellar sign**	0 (0%)	0 (0%)	20 (100%)	7 (100%)	

Abbreviations: ET-T, essential tremor without additional signs; ET-P, essential tremor with Parkinsonian signs; ET-C, essential tremor with cerebellar signs; ET-M, essential tremor with mixed Parkinsonian and cerebellar signs; CRST, Clinical Rating Scale for Tremor.

**Table 3:** (**A**) Continuous oculomotor parameters across study groups. (**B**) Frequency of oculomotor abnormalities across study groups.

Parameter	ET-T	ET-P	ET-C	ET-M	PD	DA	ET-P vs. PD	ET-C vs. DA	ET-M vs. DA
(**A**)									
**Pace-induced saccades** Amplitude (deg)	19.6 ± 4.1	18.8 ± 2.4	20.4 ± 5.1	17.2 ± 4.0	15.4 ± 4.8	17.4 ± 4.7	-	*p* = 0.025	-
**Cued saccades** Latency (ms)	453.3 ± 83.9	512.3 ± 139.8	544.0 ± 126.7	712.8 ± 126.4	603.1 ± 223.5	577.2 ± 125.0	-	-	-
**Smooth pursuit gain**	80.8 ± 7.9	69.8 ± 16.8	71.8 ± 10.1	72.0 ± 16.0	66.5 ± 21.7	73.4 ± 14.0	-	-	-
(**B**)									
**Reflexive saccades** Hypometria (%)	35.3	50.0	40.0	57.1	66.0	40.5	*p* = 0.016	-	-
**Pace-induced saccades** Hypometria (%)	5.9	0	0	0	32.0	57.1	*p* = 0.006	*p* = 0.003	*p* = 0.020
**Cued saccades** Mean error rate (%)	24.1 ± 24.2	26.4 ± 14.2	31.6 ± 15.2	37.0 ± 18.7	35.0 ± 16.7	26.5 ± 16.7	-	-	-

Abbreviations (A): ET-T, essential tremor without additional signs; ET-P, essential tremor with Parkinsonian signs; ET-C, essential tremor with cerebellar signs; ET-M, essential tremor with mixed features; PD, Parkinson’s disease; DA, degenerative ataxias. Values are presented as mean ± SD. Control data are presented in Supplementary Table S1. Abbreviations (B): ET-T, essential tremor without additional signs; ET-P, essential tremor with Parkinsonian signs; ET-C, essential tremor with cerebellar signs; ET-M, essential tremor with mixed features; PD, Parkinson’s disease; DA, degenerative ataxias. Values are presented as percentages or mean ± SD, as appropriate.

## Data Availability

The data supporting the findings of this study are available from the corresponding author upon reasonable request.

## Supplementary Materials

The following supporting information can be downloaded at: https://www.mdpi.com/article/10.3390/jcm15114054/s1, Table S1: Complete oculomotor dataset across study groups.

## Abbreviations

The following abbreviations are used in this manuscript:

ET-T	Essential tremor without additional signs
ET-P	Essential tremor with Parkinsonian signs
ET-C	Essential tremor with cerebellar signs
ET-M	Essential tremor with mixed Parkinsonian and cerebellar signs
ET-plus	Essential tremor with additional neurological signs
PD	Parkinson’s disease
DA	Degenerative ataxias
CRST	Clinical Rating Scale for Tremor
UPDRS	Unified Parkinson’s Disease Rating Scale
ICARS	International Cooperative Ataxia Rating Scale
MMSE	Mini-Mental State Examination
BDI	Beck Depression Inventory
SPG	Smooth pursuit gain
EOG	Electrooculography
