# Supplementary material for: Pace-Induced Saccades in Essential Tremor Differ from Those in Parkinson’s Disease and Degenerative Ataxias
Source: J Clin Med. 2026 May 24;15(11):4054. doi: 10.3390/jcm15114054 (PMC13258462; doi:10.3390/jcm15114054)
Supplement: Supplementary file 1 [file jcm-15-04054-s001.zip › jcm-4250398-supplementary.pdf]

Supplementary Table S1. Complete oculomotor dataset across study groups.

Type of eye movement	Parameter	ET-T	ET-P	ET-C	ET-M	PD	DA	Control group	ET-P vs. PD	ET-M vs. PD	ET-C vs. DA	ET-M vs. DA
Reflexive saccades	Latency [ms]	225.7±90.4	283.7±76.6	272.1±97.7	298.86±47.0	275.9±90.8	250.3± 61.7	227.1±49.2	ns.	ns.	ns.	ns.
	Amplitudę [deg]	19.0± 3.3	19.0±2.8	17.6±4.5	18.4±1.8	15.4±4.8	17.5±2.7	19.8±0.9	ns.	ns.	ns.	ns.
	Hypometria [%]	6 (35,3 %)	3 (50,0 %)	8 (40,0 %)	4 (57,1 %)	33 (66.0%)	17 (40.5 %)	11 (26.2 %)	χ^2=5.8 p=0.016	ns.	ns.	ns.
	Dysmetria [%]	3 (17,6 %)	1 (16,7 %)	4 (20,0 %)	1 (14,3 %)	41 (82.0 %)	32 (76.2 %)	14 (33.3 %)	ns.	ns.	ns.	ns.
	Velocity [deg/s]	529.9±62.4	520.2±93.4	472.2±117.2	488.1±51.5	462.8±122.1	486,6±110,7	486.1±104.9	ns.	ns.	ns.	ns.
Pace-induced saccades	Number	50.7±14.0	45.5±4.3	44.0±11.6	39.7±10.5	42.9±15.1	40.0±12.7	49.5±11.5	ns.	ns.	ns.	ns.
	Latency [ms]	593.5±132.0	633.2±66.0	821.7±123.2	998.4±123.2	703.6±186.7	784.6±289.8	719.1±194.3	ns.	ns.	ns.	ns.
	Amplitude [deg]	19.6±4.1	18.8±2.4	20.4±5.1	17.2±4.0	15.4±4.8	17.4±4.7	19.3±4.1	ns.	ns.	p=0.025	ns.
	Hypometria [%]	1 (5,9 %)	0	0	0	16 (32.0 %)	24 (57.1 %)	0	χ^2 =23.2 p=0.000	ns.	χ^{2Yates} =13.2 p=0.003	χ^{2Yates} = 5.7 p=0.017
	Velocity [deg/s]	528.3±118.5	515.2± 38.4	513.5±151.4	450.1±109.6	505.4±113.9	481.7±89.1	505.2±171.5	ns.	ns.	ns.	ns.
Cued saccades	Error rate [%]	24.1± 24.2	26.4±14.2	31.6±15.2	37.0± 18.7	35.0±16.7	26.5±16.7	26.8±16.1	ns.	ns.	ns.	ns.
	Latency [ms]	453.3±83.9	512.3±139.8	544.0±126.7	712.8±126.4	603.1±223.5	506.7±125.0	227,09±49,20	ns.	ns.	ns.	ns.
Smooth pursuit	SPG [%]	80.8±7.9	69.8±16.8	71.8±10.1	72.0±16.0	66.5±21,7	73.4±14,0	86.1±2.8	ns.	ns.	ns.	ns.
Saccadic intrusions	No. of cases [%]	0	0	0	2/6 33.3 %	7/13 53.8%	7/12 58.3%	1/9 11.1%	ns.	ns.	ns.	ns.

ET-T – Essential tremor without concomitant signs, ET-P – Essential tremor with parkinsonian signs, ET-C – Essential tremor with cerebellar signs, ET-M – Essential tremor with mixed parkinsonian and cerebellar signs, PD = Parkinson's disease, DA- degenerative ataxia

[1] Kruskal–Wallis one-way analysis of variance, post hoc analysis by Dunn`s test;]

[***] Pearson $\chi 2$ test
